# Supplementary material for: Species- and strain-level assessment using rrn long-amplicons suggests donor’s influence on gut microbial transference via fecal transplants in metabolic syndrome subjects
Source: Gut Microbes. 2022 May 23;14(1):2078621. doi: 10.1080/19490976.2022.2078621 (PMC9132484; doi:10.1080/19490976.2022.2078621)
Supplement: Supplemental Material [file KGMI_A_2078621_SM2704.zip › FigureS1.pdf]

➢*Streptococcus sobrinus* - rrn; length = 4,272 nt; coverage = 10 reads (low)

Description	Max Score	Total Score	Query Cover	E value	Per. Ident	Accession
Streptococcus sobrinus strain 10919 chromosome, complete genome	7284	43705	98%	0.0	97.92%	CP029491.1
Streptococcus sobrinus strain NCTC10921 genome assembly, chromosome: 1	7267	43629	98%	0.0	97.85%	LS483381.1
Streptococcus sobrinus strain NCTC12279 genome assembly, chromosome: 1	7267	43602	98%	0.0	97.85%	LS483378.1
Streptococcus sobrinus strain NIDR 6715-7 chromosome, complete genome	7267	43621	98%	0.0	97.85%	CP029560.1
Streptococcus sobrinus strain NIDR 6715-15 chromosome, complete genome	7267	43626	98%	0.0	97.85%	CP029559.1
Streptococcus sobrinus strain SL1 chromosome, complete genome	7267	43602	98%	0.0	97.85%	CP029490.1

➢*Acidaminococcus intestini* - rrn; length = 4,120 nt; coverage = 12 reads (low)

Description	Max Score	Total Score	Query Cover	E value	Per. Ident	Accession
Acidaminococcus intestini RyC-MR95, complete genome	6968	20900	98%	0.0	97.56%	CP003058.1
Acidaminococcus fermentans DSM 20731, complete genome	5729	34604	98%	0.0	92.16%	CP001859.1

➢*Eubacterium cylindroides* - rrn; length = 4,081 nt; coverage = 170 reads (mid)

Description	Max Score	Total Score	Query Cover	E value	Per. Ident	Accession
Eubacterium cylindroides T2-87 draft genome	7249	7249	98%	0.0	99.21%	FP929041.1
Faecalitalea cylindroides strain JCM 10261 16S ribosomal RNA, partial sequence	2697	2697	36%	0.0	99.14%	NR_113163.1

➢*Streptococcus thermophilus* - rrn; length = 4,112 nt; coverage = 766 reads (high)

Description	Max Score	Total Score	Query Cover	E value	Per. Ident	Accession
Streptococcus thermophilus strain GABA chromosome, complete genome	7476	44830	98%	0.0	99.83%	CP025399.1
Streptococcus thermophilus strain DGCC 7710 chromosome, complete genome	7476	37381	98%	0.0	99.83%	CP025216.1
Streptococcus thermophilus strain ST3, complete genome	7476	44830	98%	0.0	99.83%	CP017064.1
Streptococcus thermophilus strain APC151, complete genome	7476	44834	98%	0.0	99.83%	CP019935.1
Streptococcus thermophilus strain ND07, complete genome	7476	37381	98%	0.0	99.83%	CP016394.1
Streptococcus thermophilus strain KLDS SM, complete genome	7476	44847	98%	0.0	99.83%	CP016026.1
Streptococcus thermophilus strain MN-BM-A02, complete genome	7476	37200	98%	0.0	99.83%	CP010999.1
Streptococcus thermophilus strain SMQ-301, complete genome	7476	44819	98%	0.0	99.83%	CP011217.1
Streptococcus thermophilus ASCC 1275, complete genome	7476	37381	98%	0.0	99.83%	CP006819.1
Streptococcus thermophilus JIM 8232 complete genome	7476	44810	98%	0.0	99.83%	FR875178.1
Streptococcus thermophilus LMD-9, complete genome	7476	44819	98%	0.0	99.83%	CP000419.1
Streptococcus thermophilus strain EPS chromosome, complete genome	7472	44830	98%	0.0	99.80%	CP025400.1

➢*Akkermansia muciniphila* - rrn; length = 4,306 nt; coverage = 1000 reads (high)

Description	Max Score	Total Score	Query Cover	E value	Per. Ident	Accession
Akkermansia muciniphila strain EB-AMDK-3 chromosome, complete genome	7755	23258	98%	0.0	99.53%	CP024738.1
Akkermansia muciniphila strain EB-AMDK-4 chromosome, complete genome	7751	23254	98%	0.0	99.51%	CP024740.1
Akkermansia muciniphila strain CBA5201 chromosome, complete genome	7751	23254	98%	0.0	99.51%	CP033388.1
Akkermansia muciniphila strain EB-AMDK-7 chromosome, complete genome	7751	23248	98%	0.0	99.51%	CP025823.1
Akkermansia muciniphila ATCC BAA-835, complete genome	7751	23254	98%	0.0	99.51%	CP001071.1
Akkermansia muciniphila strain EB-AMDK-16 chromosome, complete genome	7749	23243	98%	0.0	99.51%	CP025831.1
Akkermansia muciniphila strain H2 chromosome	7745	22576	98%	0.0	99.48%	CP010553.1

➢*Faecalibacterium prausnitzii* - rrn; length = 3,986 nt; coverage = 1000 reads (high)

Description	Max Score	Total Score	Query Cover	E value	Per. Ident	Accession
Faecalibacterium prausnitzii strain Indica chromosome, complete genome	6916	45825	99%	0.0	98.21%	CP023819.1
Faecalibacterium prausnitzii strain A2165 chromosome, complete genome	6902	41296	99%	0.0	98.14%	CP022479.1
Faecalibacterium prausnitzii strain APC918/95b chromosome, complete genome	6859	41078	99%	0.0	97.96%	CP030777.1
Faecalibacterium prausnitzii strain 942/30-2 chromosome, complete genome	6735	40347	99%	0.0	97.39%	CP026548.1
