# Supplementary material for: Species- and strain-level assessment using rrn long-amplicons suggests donor’s influence on gut microbial transference via fecal transplants in metabolic syndrome subjects
Source: Gut Microbes. 2022 May 23;14(1):2078621. doi: 10.1080/19490976.2022.2078621 (PMC9132484; doi:10.1080/19490976.2022.2078621)
Supplement: Supplemental Material [file KGMI_A_2078621_SM2704.zip › FigureS2.pdf]

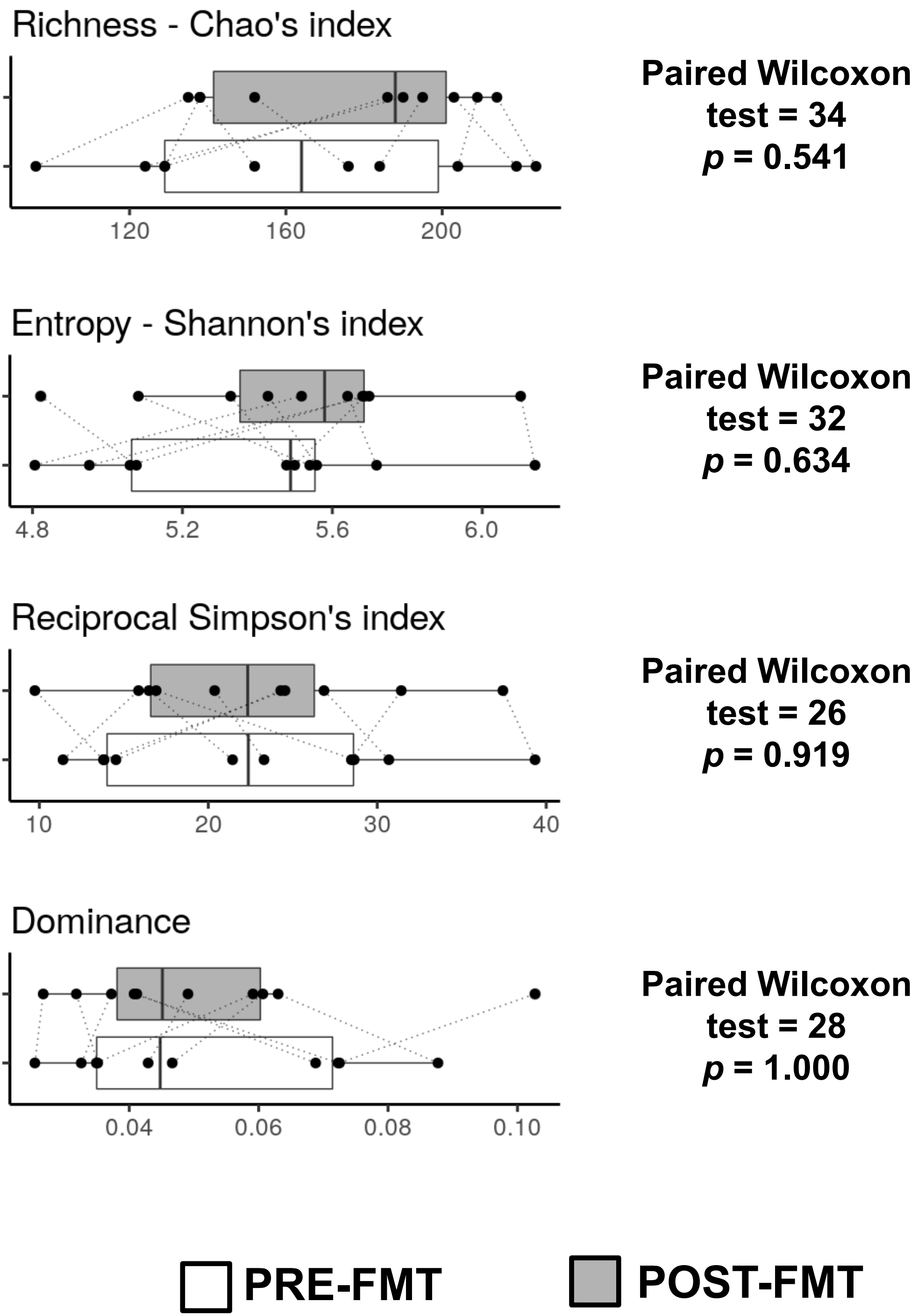
Richness - Chao's index
120
160
200
Paired Wilcoxon
test = 34
p = 0.541
Entropy - Shannon's index
4.8
5.2
5.6
6.0
Paired Wilcoxon
test = 32
p = 0.634
Reciprocal Simpson's index
10
20
30
40
Paired Wilcoxon
test = 26
p = 0.919
Dominance
0.04
0.06
0.08
0.10
Paired Wilcoxon
test = 28
p = 1.000
PRE-FMT
POST-FMT
