# Supplementary material for: Species- and strain-level assessment using rrn long-amplicons suggests donor’s influence on gut microbial transference via fecal transplants in metabolic syndrome subjects
Source: Gut Microbes. 2022 May 23;14(1):2078621. doi: 10.1080/19490976.2022.2078621 (PMC9132484; doi:10.1080/19490976.2022.2078621)
Supplement: Supplemental Material [file KGMI_A_2078621_SM2704.zip › FigureS3.pdf]

Parabacteroides merdae SNV-3527 as query (pos 3470-3570)

Sequences producing significant alignments — Download | New Select columns | Show 100

select all — 5 sequences selected — GenBank | Graphics | Distance tree of results | New MSA Viewer

	Description	Scientific Name	Max Score	Total Score	Query Cover	E value	Per. Ident	Acc. Len	Accession
☑	Parabacteroides merdae strain CL06T03C08 chromosome, complete genome	Parabacteroides merdae	187	1125	100%	1e-43	100.00%	4697288	CP072229.1
☑	Uncultured bacterium clone LM0ACA6ZA05RM1 genomic sequence	uncultured bacterium	182	182	100%	5e-42	99.01%	706	EU066707.1
☑	Uncultured bacterium clone LM0ACA6ZD02FM1 genomic sequence	uncultured bacterium	182	182	100%	5e-42	99.01%	710	EU063890.1
☑	Uncultured bacterium clone LM0ACA4ZC10FM1 genomic sequence	uncultured bacterium	182	182	100%	5e-42	99.01%	585	EU063717.1
☑	Bacteroides merdae 23S ribosomal RNA gene, partial sequence	Parabacteroides merdae ATCC 43184	182	182	100%	5e-42	99.01%	574	AY155595.1

Download | GenBank | Graphics | Sort by: E value

Parabacteroides merdae strain CL06T03C08 chromosome, complete genome

Sequence ID: CP072229.1 Length: 4697288 Number of Matches: 6

Range 1: 37534 to 37634 GenBank Graphics — ▼ Next Match ▲ Previous Match

Score	Expect	Identities	Gaps	Strand
187 bits(101)	1e-43	101/101(100%)	0/101(0%)	Plus/Minus

```
Query  1      CCTGATTACGTCCATTTTGCCTTGTGCAAGCACGCGGCATACTATCAAGGTCGACTCTCC  60
              ||||||||||||||||||||||||||||||||||||||||||||||||||||||||||||
Sbjct  37634  CCTGATTACGTCCATTTTGCCTTGTGCAAGCACGCGGCATACTATCAAGGTCGACTCTCC  37575

Query  61     CTGCGGATTTGCCTACAGGAATCTACATCTACACTCTTCAA  101
              |||||||||||||||||||||||||||||||||||||||||
Sbjct  37574  CTGCGGATTTGCCTACAGGAATCTACATCTACACTCTTCAA  37534
```

Strain CL06T03C08 genotype A3527

Download | GenBank | Graphics

Uncultured bacterium clone LM0ACA6ZA05RM1 genomic sequence

Sequence ID: EU066707.1 Length: 706 Number of Matches: 1

Range 1: 210 to 310 GenBank Graphics — ▼ Next Match ▲ Previous Match

Score	Expect	Identities	Gaps	Strand
182 bits(98)	5e-42	100/101(99%)	0/101(0%)	Plus/Minus

```
Query  1    CCTGATTACGTCCATTTTGCCTTGTGCAAGCACGCGGCATACTATCAAGGTCGACTCTCC  60
            |||||||||||||||||||||||||||||||||||||||||| |||||||||||||||||
Sbjct  310  CCTGATTACGTCCATTTTGCCTTGTGCAAGCACGCGGCATACTGTCAAGGTCGACTCTCC  251

Query  61   CTGCGGATTTGCCTACAGGAATCTACATCTACACTCTTCAA  101
            |||||||||||||||||||||||||||||||||||||||||
Sbjct  250  CTGCGGATTTGCCTACAGGAATCTACATCTACACTCTTCAA  210
```

Strain LM0ACA6ZA05RM1 genotype G3527

Download | GenBank | Graphics

Uncultured bacterium clone LM0ACA6ZD02FM1 genomic sequence

Sequence ID: EU063890.1 Length: 710 Number of Matches: 1

Range 1: 316 to 416 GenBank Graphics — ▼ Next Match ▲ Previous Match

Score	Expect	Identities	Gaps	Strand
182 bits(98)	5e-42	100/101(99%)	0/101(0%)	Plus/Plus

```
Query  1    CCTGATTACGTCCATTTTGCCTTGTGCAAGCACGCGGCATACTATCAAGGTCGACTCTCC  60
            |||||||||||||||||||||||||||||||||||||||||| |||||||||||||||||
Sbjct  316  CCTGATTACGTCCATTTTGCCTTGTGCAAGCACGCGGCATACTGTCAAGGTCGACTCTCC  375

Query  61   CTGCGGATTTGCCTACAGGAATCTACATCTACACTCTTCAA  101
            |||||||||||||||||||||||||||||||||||||||||
Sbjct  376  CTGCGGATTTGCCTACAGGAATCTACATCTACACTCTTCAA  416
```

Strain LM0ACA6ZD02FM1 genotype G3527

Download | GenBank | Graphics

Uncultured bacterium clone LM0ACA4ZC10FM1 genomic sequence

Sequence ID: EU063717.1 Length: 585 Number of Matches: 1

Range 1: 396 to 496 GenBank Graphics — ▼ Next Match ▲ Previous Match

Score	Expect	Identities	Gaps	Strand
182 bits(98)	5e-42	100/101(99%)	0/101(0%)	Plus/Plus

```
Query  1    CCTGATTACGTCCATTTTGCCTTGTGCAAGCACGCGGCATACTATCAAGGTCGACTCTCC  60
            |||||||||||||||||||||||||||||||||||||||||| |||||||||||||||||
Sbjct  396  CCTGATTACGTCCATTTTGCCTTGTGCAAGCACGCGGCATACTGTCAAGGTCGACTCTCC  455

Query  61   CTGCGGATTTGCCTACAGGAATCTACATCTACACTCTTCAA  101
            |||||||||||||||||||||||||||||||||||||||||
Sbjct  456  CTGCGGATTTGCCTACAGGAATCTACATCTACACTCTTCAA  496
```

Strain LM0ACA4ZC10FM1 genotype G3527

Download | GenBank | Graphics

Bacteroides merdae 23S ribosomal RNA gene, partial sequence

Sequence ID: AY155595.1 Length: 574 Number of Matches: 1

Range 1: 391 to 491 GenBank Graphics — ▼ Next Match ▲ Previous Match

Score	Expect	Identities	Gaps	Strand
182 bits(98)	5e-42	100/101(99%)	0/101(0%)	Plus/Minus

```
Query  1    CCTGATTACGTCCATTTTGCCTTGTGCAAGCACGCGGCATACTATCAAGGTCGACTCTCC  60
            |||||||||||||||||||||||||||||||||||||||||| |||||||||||||||||
Sbjct  491  CCTGATTACGTCCATTTTGCCTTGTGCAAGCACGCGGCATACTGTCAAGGTCGACTCTCC  432

Query  61   CTGCGGATTTGCCTACAGGAATCTACATCTACACTCTTCAA  101
            |||||||||||||||||||||||||||||||||||||||||
Sbjct  431  CTGCGGATTTGCCTACAGGAATCTACATCTACACTCTTCAA  391
```

Strain ATCC43184 genotype G3527
