# Supplementary material for: Species- and strain-level assessment using rrn long-amplicons suggests donor’s influence on gut microbial transference via fecal transplants in metabolic syndrome subjects
Source: Gut Microbes. 2022 May 23;14(1):2078621. doi: 10.1080/19490976.2022.2078621 (PMC9132484; doi:10.1080/19490976.2022.2078621)

Albacore v2.1.3	Guppy v5.0.11

Run1 – Per base sequence quality

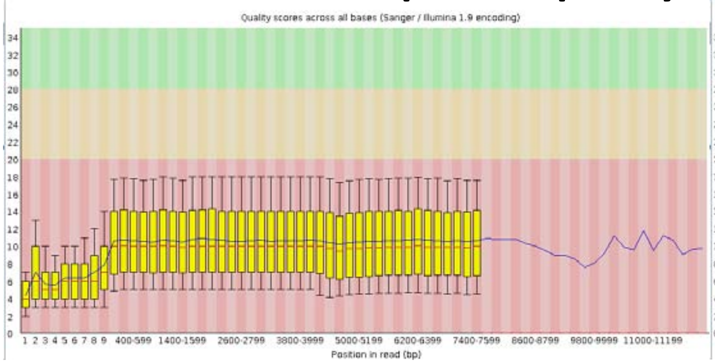

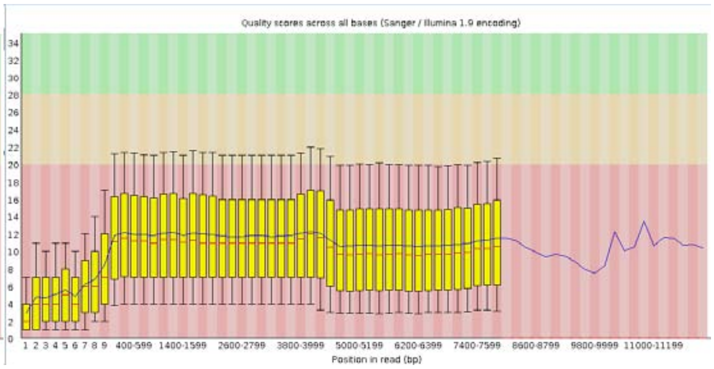

Run1 – Per sequence quality scores

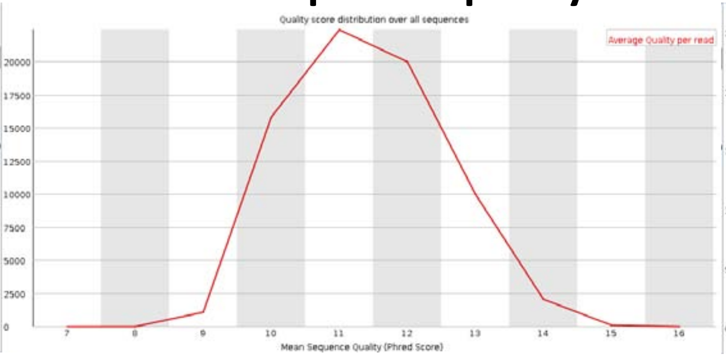

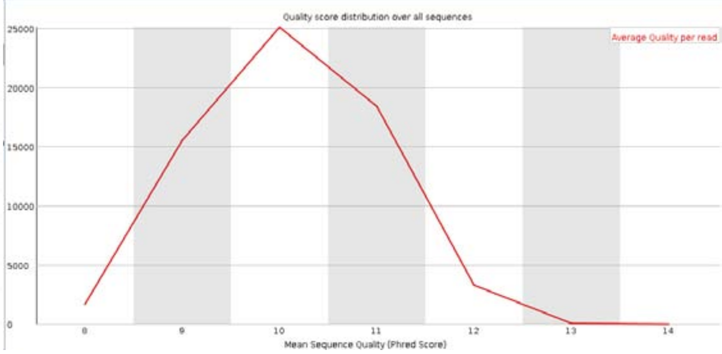

Run1 – Per base sequence content

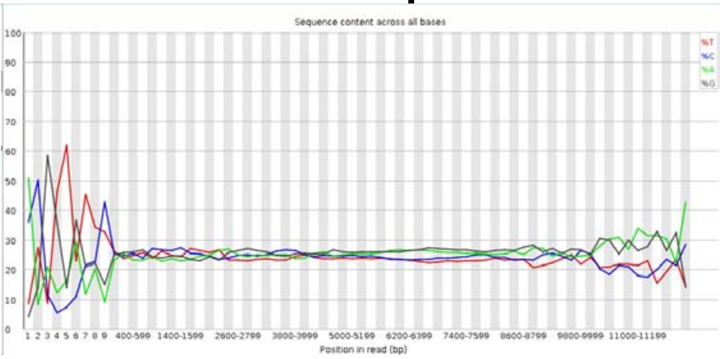

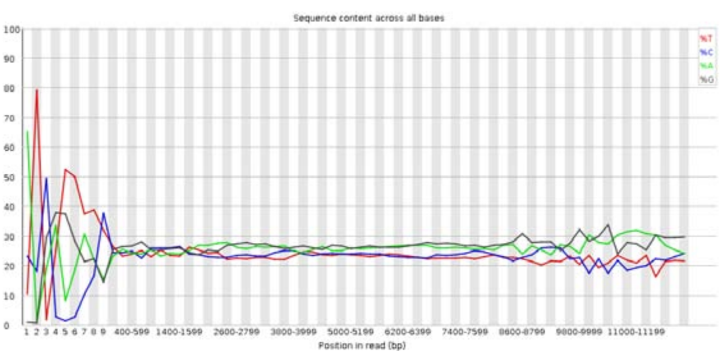

Run1 – Sequence length distribution

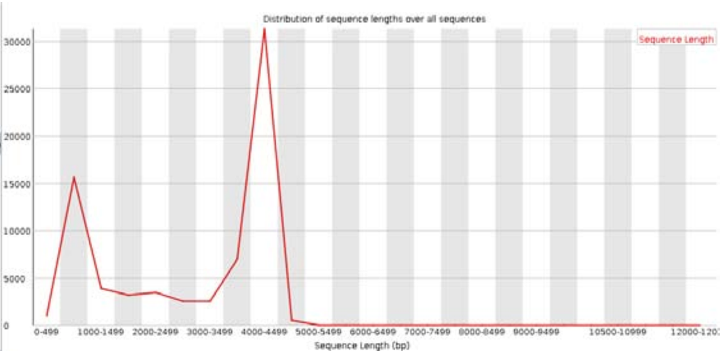

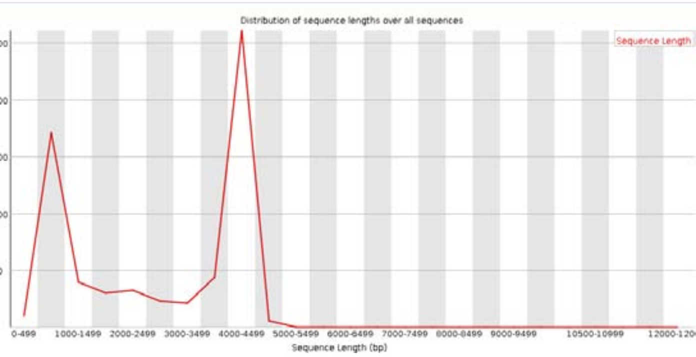

Albacore v2.1.3 | Guppy v5.0.11

Run2 – Per base sequence quality

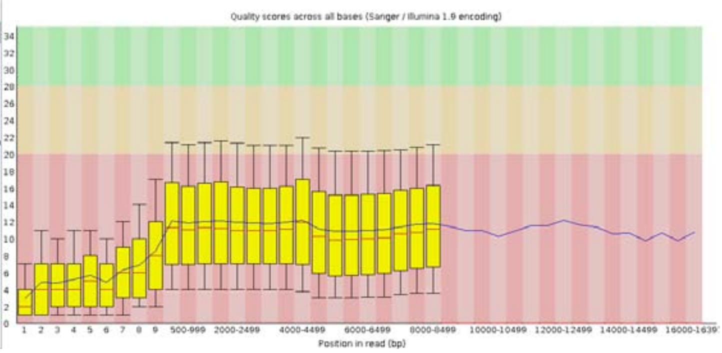

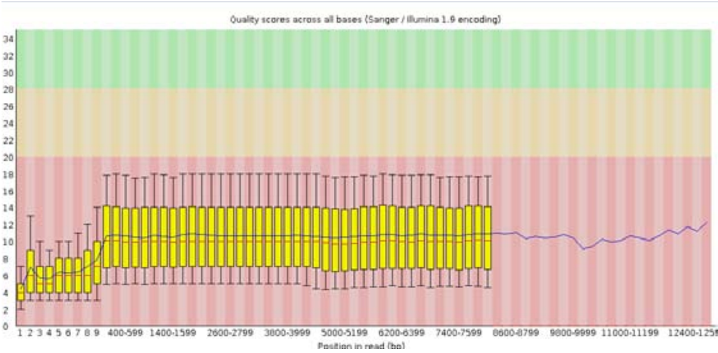

Run2 – Per sequence quality scores

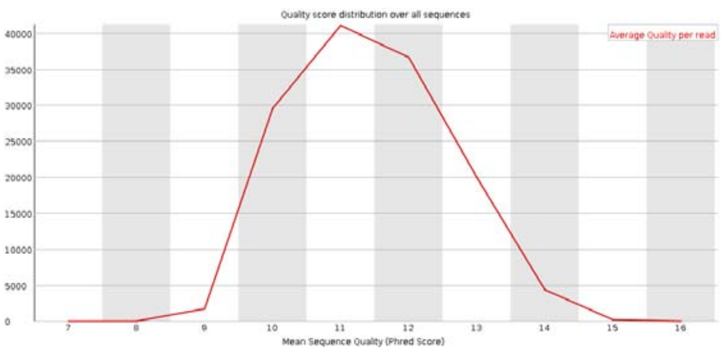

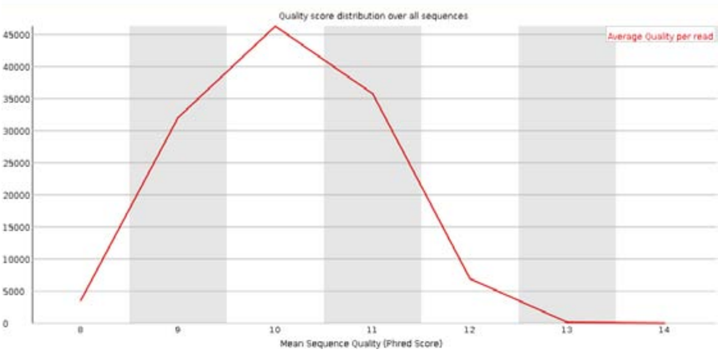

Run2 – Per base sequence content

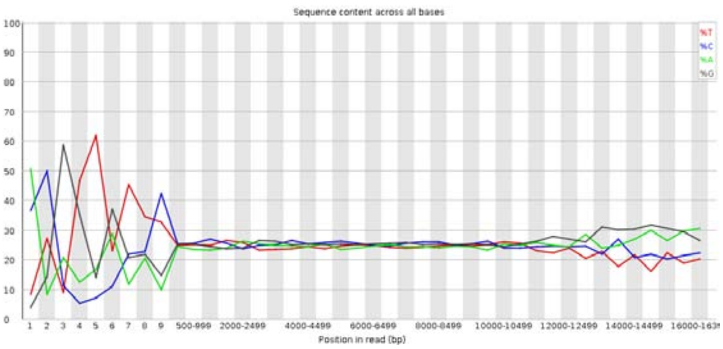

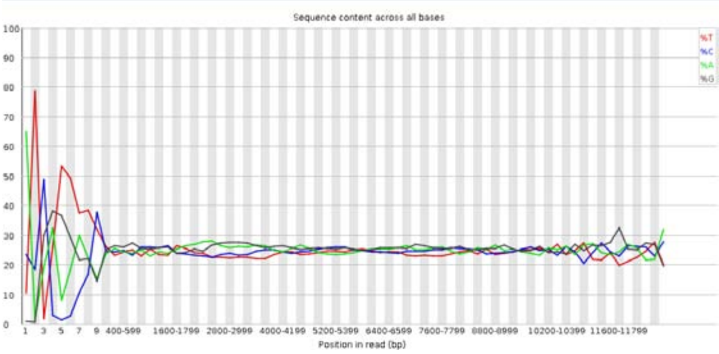

Run2 – Sequence length distribution

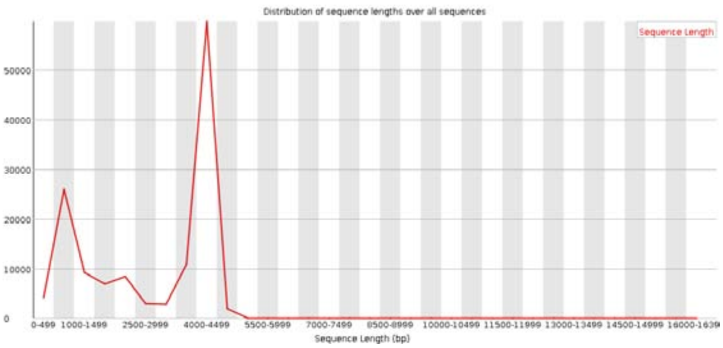

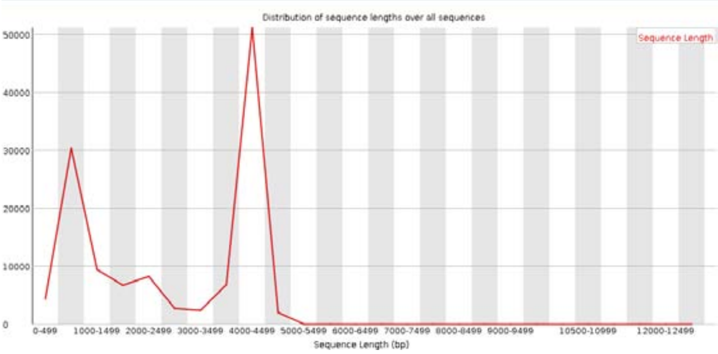

Albacore v2.1.3	Guppy v5.0.11

Run3 – Per base sequence quality

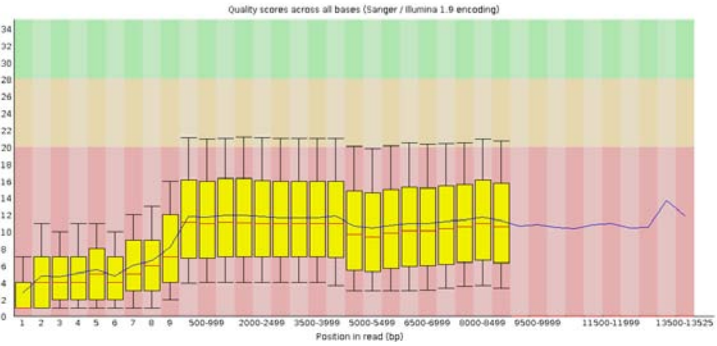

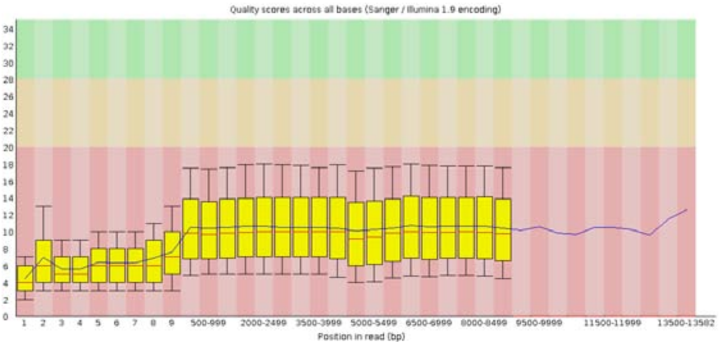

Run3 – Per sequence quality scores

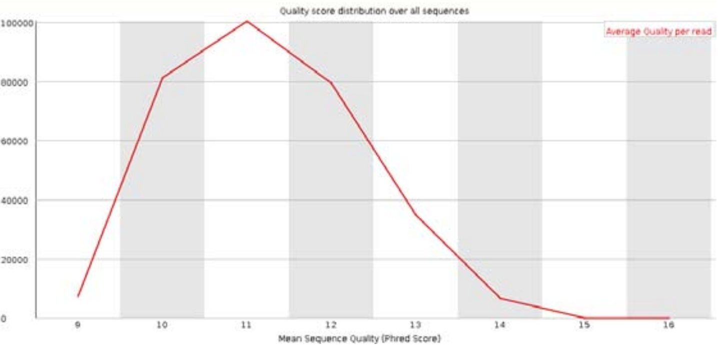

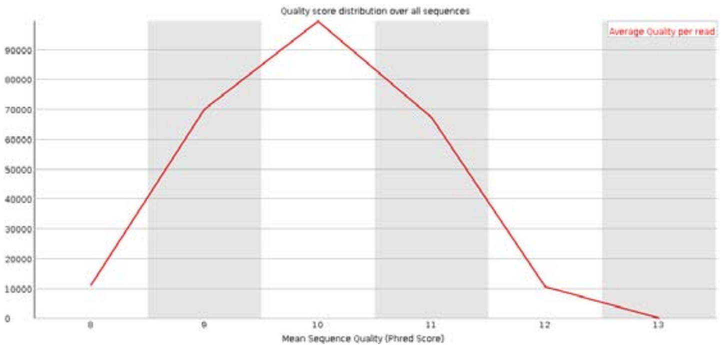

Run3 – Per base sequence content

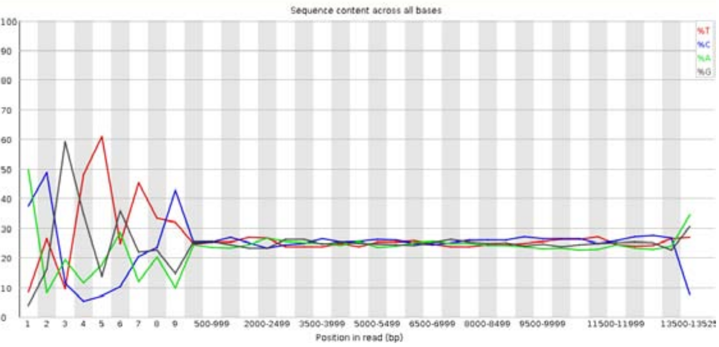

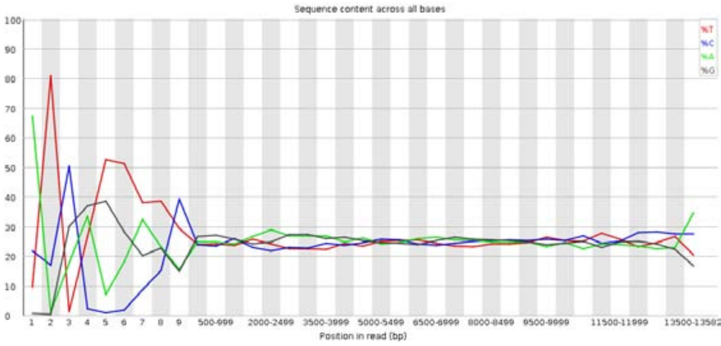

Run3 – Sequence length distribution

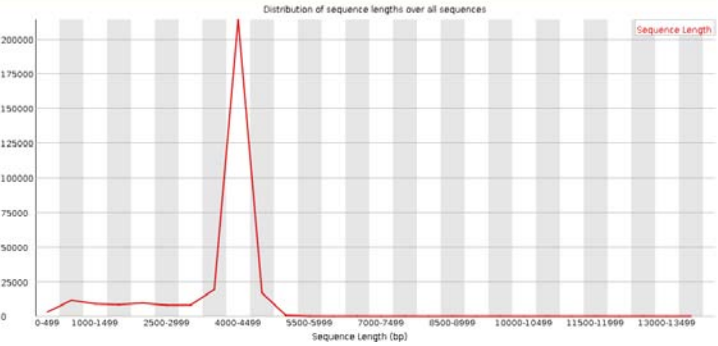

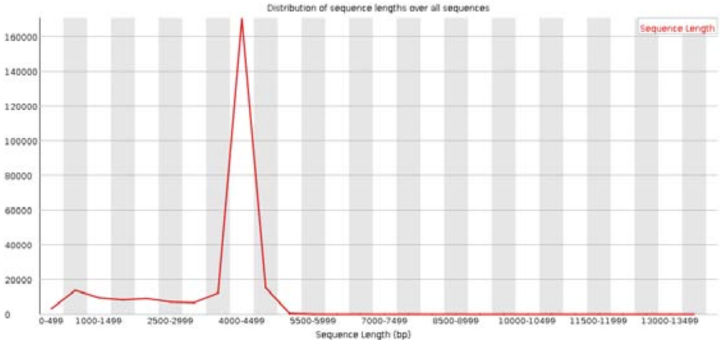

Supplement: Supplemental Material [file KGMI_A_2078621_SM2704.zip › FigureS4.pdf]
